# Examining the construct and known-group validity of a composite endpoint for The Older Persons and Informal Caregivers Survey Minimum Data Set (TOPICS-MDS); A large-scale data sharing initiative

**DOI:** 10.1371/journal.pone.0173081

**Published:** 2017-03-15

**Authors:** Cynthia S. Hofman, Jennifer E. Lutomski, Han Boter, Bianca M. Buurman, Anton J. M. de Craen, Rogier Donders, Marcel G. M. Olde Rikkert, Peter Makai, René J. F. Melis, TOPICS-MDS research consortium

**Affiliations:** 1 Department of Geriatric Medicine (HP 925), Radboud University Medical Center, Nijmegen, The Netherlands; 2 Department for Health Evidence (HP133), Radboud University Medical Center, Nijmegen, the Netherlands; 3 Vilans, Centre of Expertise for Long-Term Care, Utrecht, the Netherlands; 4 Anu Research Centre, University College Cork, Cork, Ireland; 5 Department of Epidemiology (FA41), University of Groningen, University Medical Centre Groningen, Groningen, the Netherlands; 6 Department of Internal Medicine, Section of Geriatric Medicine (F4-108), Academic Medical Center, Amsterdam, the Netherlands; 7 Department of Gerontology and Geriatrics (C2-R), Leiden University Medical Centre, Leiden, the Netherlands; Public Library of Science, FRANCE

## Abstract

**Background:**

Preference-weighted multi-faceted endpoints have the potential to facilitate comparative effectiveness research that incorporates patient preferences. The Older Persons and Informal Caregivers Survey—Composite endpoint (TOPICS-CEP) is potentially a valuable outcome measure for evaluating interventions in geriatric care as it combines multiple outcomes relevant to older persons in a single metric. The objective of this study was to validate TOPICS-CEP across different study settings (general population, primary care and hospital).

**Methods:**

Data were extracted from TOPICS Minimum Dataset (MDS), a pooled public-access national database with information on older persons throughout the Netherlands. Data of 17,603 older persons were used. Meta-correlations were performed between TOPICS-CEP indexed scores, EuroQol5-D utility scores and Cantril’s ladder life satisfaction scores. Mixed linear regression analyses were performed to compare TOPICS-CEP indexed scores between known groups, e.g. persons with versus without depression.

**Results:**

In the complete sample and when stratified by study setting TOPICS-CEP and Cantril’s ladder were moderately correlated, whereas TOPICS-CEP and EQ-5D were highly correlated. Higher mean TOPICS-CEP scores were found in persons who were: married, lived independently and had an education at university level. Moreover, higher mean TOPICS-CEP scores were found in persons without dementia, depression, and dizziness with falls, respectively. Similar results were found when stratified by subgroup.

**Conclusion:**

This study supports that TOPICS-CEP is a robust measure which can potentially be used in broad settings to identify the effect of intervention or of prevention in elderly care.

## Introduction

Aging of the population has a major impact on the organization and delivery of healthcare. The shift from acute to chronic illnesses and the expected shortage of healthcare workers will be of particular importance.[1] To ensure high quality care for older persons, the evaluation and monitoring of three aspects of health care delivery need to be regularly evaluated: structure, process, and outcomes.[2] However, comparing outcomes in older persons is challenging. Firstly, the health states of older persons are complex, as older individuals often present different combinations of chronic multi-morbidity and functional limitations.[3] Secondly, interventions often influence a broad range of health domains both directly and indirectly. For example, occupational therapy aims to enable people who have physical restrictions to achieve greater independence. By engaging in meaningful social activities, health and psychological wellbeing are also indirectly and positively influenced.[4] Thus, occupational therapy can improve both physical and mental wellbeing. The two obstacles can be circumvented if the important outcome parameters are collected and combined into a preference-weighted composite endpoint (CEP) for health and wellbeing.[5, 6] Preference-weighed refers to placing value judgments on the components included in the CEP. These weights reflect the relative importance of each component when compared with an anchor, such as perfect health, quality of life, or general wellbeing. The fact that most definitions of quality of care consistently stress the importance of patient-centredness underlines the necessity of using preference-weights to combine multidimensional items if the aim is to measure the value of care. Hence, in order to assess quality of healthcare, the outcome measure used needs to reflect the *value* of the change accomplished according to the patient.

In 2008, the Dutch Care for the Elderly Programme was commissioned by the Ministry of Health, Welfare and Sport with the guiding principles of improving care, quality of life, and self-management among older persons. As part of this Programme, The Older Persons and Informal Caregivers Survey Minimum Data Set (TOPICS-MDS) was developed to uniform collection of outcome measures.[7] To promote comparability between research studies, a preference-weighted CEP was established for TOPICS*-MDS* based on the health state valuations of older persons and informal caregivers. This CEP (referred to as TOPICS-CEP) was designed as a multi-faceted outcome measure applying weights derived from older persons’ priorities for different outcomes to assist in the evaluation of interventions in older persons by measuring health-realted quality of life (HR-QOL).[8]

TOPICS-CEP has been previously developed using a vignette study in which 200 persons participated. The vignettes described eight TOPICS-MDS outcomes of older persons (morbidity, functional limitations, emotional wellbeing, pain experience, cognitive functioning, social functioning, self-perceived health and self-perceived quality of life) and the raters assessed the general wellbeing (GWB) of these vignette cases on a numeric rating scale (0–10). Mixed linear regression analyses were used to derive the preference weights of the TOPICS-MDS outcomes (dependent variable: GWB scores; fixed factors: the eight outcomes; unstandardized coefficients: preference weights).[8] The aim of this current study was to determine TOPICS-CEP’s convergent and known-groups validity in large heterogeneous samples of older persons aged 65 years and older and across general population, primary care and hospital setting.

## Methods

### Data source

Data were derived from TOPICS-MDS (www.topics-mds.eu), which is a public data repository designed to capture essential information on the physical and mental wellbeing of older persons and informal caregivers in the Netherlands. A detailed description of TOPICS-MDS has been presented elsewhere.[7] Briefly, TOPICS-MDS consists of pooled data from various research projects which differ across study design, sampling framework, and inclusion criteria. All data were cleaned locally using a standardized protocol. Anonymized individual-level data were then submitted to a central institution (Radboud University Medical Center, Nijmegen, the Netherlands) for further validation checks and creation of the pooled dataset. Since various research projects submit information to TOPICS-MDS, the database is dynamic in nature and thus regularly updated with new observations.

Our present analysis uses the first version of the dataset available as of January 2013 and is based on 41 studies with data available on 32,310 older persons. Studies which omitted TOPICS-CEP data points by design were excluded from this study. This, resulted in a final study sample of 17,603 older persons.

TOPICS-MDS is a fully anonymized dataset available for public access, and therefore this analysis was exempt from ethical review (Radboud University Medical Center Ethical Committee review reference number: CMO: 2012/120).

### Measures

#### TOPICS-CEP

TOPICS-CEP score is a preference-weighted index ranging from 0 (worst possible state) to 10 (best possible state) that combines 42 data points representing eight domains: morbidities (list of 17 pre-defined conditions widely used in the Netherlands),[9] functional limitations (Katz index of independence),[10] emotional wellbeing (mental health subscale of the RAND-36),[11] pain experience (pain dimension of the EQ-5D),[12] cognitive problems (cognition dimension of the EQ-5D+C),[12] social functioning (item 10 from the RAND-36),[11] self-perceived health (item from the RAND-36)[11] and self-perceived quality of life (phrasing similar to self-perceived health item from the RAND-36).[11] The components vary in scale range and preference weight. More detailed information about TOPICS-CEP, including a description of the data points, can be found elsewhere.[13] Briefly, TOPICS-CEP score is calculated in four steps. Firstly, data points are coded in the same direction by means of reversed scoring. Secondly, all items that belong to the same health domain are aggregated into one component. Thus, 17 morbidity items are combined into the component *number of morbidities*, 15 items regarding functional limitations into *number of functional limitations*, and 5 emotional well-being items into *raw emotional well-being score*. Thirdly, a raw TOPICS-CEP score is calculated by means of applying the preference weights for the Dutch population aged 65 years and over.

$$\begin{array}{ll} Raw\;TOPICS & -CEP\;score\\ & =9.00\;\left(intercept\right)-\left(0.18*morbidities\right)-\left(0.12*functional\;limitations\right)\\ & -\left(0.03*emotional\;wellbeing\right)-\left(0.03*pain\;experience\right)\\ & -\left(0.14*cognitive\;problems\right)-\left(0.01*social\;functioning\right)\\ & -\left(0.17*self\;perceived\;health\right)-\left(0.02*self\;perceived\;quality\;of\;life\right) \end{array}$$

Finally, the raw TOPICS-CEP score is transformed into an indexed score (referred to as TOPICS-CEP score) ranging 0 to10.

$$TOPICS-CEP\;score=\left[\frac{\left(raw\;TOPICS\;CEP\;score-Minimum\;raw\;TOPICS\;CEP\;score\right)}{Raw\;score\;range}\right]*10$$

In this current study, only missing data points were allowed for the aggregated TOPICS-CEP components morbidities, functional limitations and emotional wellbeing. The thresholds used were less than 5 missing values for morbidities and functional limitations respectively, and less than 2 missing values for emotional wellbeing. Estimation for these data points was done by pro-rating the score. For instance, the component functional limitations includes 15 items and the scale range is 0 to 15; when 12 items are answered and the sum of the answered items is 6, then score pro-rating = [(6/12) x 15] = 7.5.

#### Other measures

The Cantril’s life satisfaction score is a one-dimensional index ranging from 0 (completely unsatisfied with life) to 10 (completely satisfied with life) and measures self-perceived general QOL.[14] We used a modified version of Cantril’s self anchoring ladder where respondents were asked to rate their present life on a scale between zero and ten.

The EuroQol-5D (EQ-5D) utility score measures health related QOL (HRQOL).[15] Five dimensions (mobility, self-care, daily activities, pain and discomfort, anxiety and depression) with three levels each (1 = no problems, 2 = moderate problems, and 3 = extreme problems) are combined into one utility score by means of applying the scoring values for the Dutch population.[15] The EQ-5D utility score ranges from -0.33 to 1.00 where a score of less than zero is indicative of a health state worse than death.[15] The item regarding pain from the EQ-5D is included in the TOPICS-CEP, thus minimizing the overlap between the two measurements with one single item. Hence, we do not expect this to influence the correlation between the two measurements.

Socio-demographic characteristics included in our analyses were marital status, living arrangements, and education level. Included clinical data points were dementia, depression, and dizziness with falls.

### Convergent validity

Convergent validity refers to how closely a measure is related to other measure of the same construct. We examined convergent validity of TOPICS-CEP score with the Cantril’s life satisfaction score and the EQ-5D utility score respectively, [14, 15] Convergent validity is determined by the correlation between the outcome measures.

#### Hypotheses

We anticipated a moderate positive correlation between TOPICS-CEP score and the Cantril’s life satisfaction score, because TOPICS-CEP intends to measure a broader concept than self-perceived general QOL. In contrast, we expected a strong positive correlation between TOPICS-CEP score and the EQ-5D utility score as both measures combines multiple outcomes, however they do have a different score range [TOPICS-CEP: 0–10 versus EQ-5D: -0.33–1.0].

### Known-group validity

After examining the convergent validity, we examined whether groups with different marital status, living arrangements, education levels and the presence or absence of the chronic conditions dementia, depression, and dizziness with falls could be distinguished based on their TOPICS-CEP scores. Thus, we assessed whether baseline TOPICS-CEP scores were significantly different between groups.

#### Hypotheses

We expected higher scores in persons who are married or cohabiting compared to widowers and in those who live with others (e.g. partner or children) compared to those who live alone because long lasting relationships positively influences (mental) health status.[16] Moreover, other previous studies have shown that those who were single, divorced, or bereaved showed higher morbidity compared with those who were married or cohabiting which negatively influence TOPICS-CEP scores. [17] Similarly, we expected to find higher scores in older persons living independently compared to those living in an institutionalized facility. This is largely due to institutionalized older persons often require more assistance with daily activities and thus may fear their loss of independence, control and dignity.[18] Furthermore, we anticipated to find lower scores in subgroups of persons with dementia, depression, or dizziness with falls than in persons without these conditions. Such conditions have wide-reaching effects and would likely negatively impact other domains included in TOPICS-CEP.[19–21] Moreover, numerous studies have shown the inverse relationship between chronic conditions and QOL.[22]

### Generalizability

To examine whether the validation results for TOPICS-CEP are generalizable across different settings, we performed additional analyses using the complete study sample as well as stratified across three major study settings: older persons in primary care setting, general older population, and hospitalized older persons.

### Analyses

Feasibility was assessed by calculating the number of missing values for TOPICS-CEP. Floor and ceiling effects were assessed by reporting the proportion of respondents with minimum and maximum TOPICS-CEP scores, respectively. A floor or ceiling effect of 15% was considered the maximum acceptable.[23]

Since TOPICS-MDS is a pooled dataset, we applied meta-analytical techniques to account for clustering within individual research projects. Pearson’s correlations were used to examine convergent validity between TOPICS-CEP, Cantril’s life satisfaction scale, and EQ-5D utility score within each study. To calculate the pooled correlation coefficients random effects meta-correlations were performed.[24] Correlations below 0.3 were referred to as weak, between 0.3 and 0.5 as moderate, and above 0.5 as strong.[25]

Known group validity was examined by determining significant differences in mean TOPICS-CEP index scores. Mixed linear regression analyses were used to compare the scores between groups and to examine whether differences between groups were still present when adjusted for age and gender. To account for clustering within individual research projects the models included random intercepts for project. The models were constructed based on *a priori* expectations. Differences between parameter estimates smaller than 15% were considered to be acceptable. Analyses were performed using SPSS version 20.0 (SPSS IBM, New York, USA) and the Meta package in R (Foundation for statistical computing, Vienna, Austria).[24]

## Results

### Sample characteristics

Data from 17,603 older persons from 28 projects were included in this study. The majority of the study sample were women (N = 10,817, 61.5%) and the mean (±SD) age was 79 (7) years. Overall, the sample consisted of 7,849 (44.9%) subjects living independently with others, 8,187 (46.7%) were married or cohabiting, and 7,965 (46.7%) had a secondary education level. The conditions dementia, depression, and dizziness with falls were present in; 962 (5.6%), 1,558 (9.1%), and 2,495 (14.6%) subjects of the study sample respectively. The socio-demographic distribution within the subgroups (primary care (N = 11,892), general population (N = 3,331), and hospital (N = 1,534)) were similar to the combined sample.

### Outcomes

Of the 17,603 participants, the majority had no missing data points for **TOPICS-CEP**: 88.7% (N = 15,612), **Cantril’s ladder**: 91.9% (N = 16,178) and **EQ-5D**: 96.6% (N = 17,006). The means (±SD; minimum and maximum scores achieved) were **TOPICS-CEP:** 7.37 (1.23; 1.88–10.0); **Cantril’s ladder:** 7.12 (1.40;0.0–10.0); and **EQ-5D:** 0.63 (0.29; -0.33–1.0). Table 1 gives an overview of the mean (±SD) scores and floor and ceiling effects for the complete sample and stratified by subgroup. The highest values possible for TOPICS-CEP, Cantril’s ladder, and EQ-5D was reported for 18 (0.1%), 379 (2.2%), and 2,009(11.4%) older persons respectively. For each outcome measure, the lowest value possible was calculated for less than 1% of the subjects. When stratified by subgroup the mean (±SD) scores showed similar patterns. For each outcome measure the lowest value possible was achieved by less than 1% of the older persons whereas the highest possible value for EQ-5D was calculated for 19.6% (N = 653) and 13.7% (N = 210) of the older persons sampled from the general population and hospital respectively.

**Table 1:** The mean (±SD) scores and floor and ceiling effects for the complete sample and stratified by subgroup.

	Mean (SD)	Floor N (%)	Ceiling N (%)
**Complete study sample** (N = 17,603)			
TOPICS-CEP	7.37 (1.23)	0 (0.0)	18 (0.1)
Cantril's ladder	7.12 (1.40)	22 (0.1)	379 (2.2)
EQ-5D	0.63 (0.29)	0 (0.0)	2009 (11.4)
**Subgroups by study setting**			
**Primary care setting** (N = 11,892)			
TOPICS-CEP	7.44 (1.15)	0 (0.0)	12 (0.1)
Cantril's ladder	7.11 (1.42)	17 (0.1)	257 (2.2)
EQ-5D	0.61 (0.28)	0 (0.0)	1100 (9.2)
**General population** (N = 3,331)			
TOPICS-CEP	7.37 (1.40)	0 (0.0)	5 (0.2)
Cantril's ladder	7.07 (1.34)	4 (0.1)	47 (1.4)
EQ-5D	0.72 (0.26)	0 (0.0)	653 (19.6)
**Hospital** (N = 1,534)			
TOPICS-CEP	7.48 (1.20)	0 (0.0)	1 (0.1)
Cantril's ladder	7.36 (1.35)	1 (0.1)	49 (3.2)
EQ-5D	0.61 (0.30)	0 (0.0)	210 (13.7)

Notes:

TOPICS-CEP is a HR-QOL tool, with a range of 0 to 10.

Cantril’s ladder is a genral QOL tool, with a range of 0 to 10.

EQ-5D is a 5-dimensional HR-QOL tool, range -0.33 to 1.00.

### Convergent validity

Table 2 gives an overview of the meta-correlation coefficients and the 95% CI. Expectedly, TOPICS-CEP and Cantril’s ladder were moderately correlated in the overall sample and subgroups **Complete sample:** r = 0.43; **Primary care:** r = 0.41; **General population:** r = 0.50; **Hospital:** r = 0.43. In comparison, TOPICS-CEP and the EQ-5D were highly correlated [**Complete sample:** r = 0.63; **Primary care:** r = 0.60; **General population:** r = 0.71; **Hospital:** r = 0.57].

**Table 2:** Meta-correlation coefficients and the 95% CI of the outcome measures TOPICS-CEP, Cantril’s ladder, and EQ-5D utility score for the complete study sample and stratified by subgroup.

	TOPICS-CEP		Cantril's ladder	
	r	95% CI	r	95% CI
**Complete study sample** (N = 17,603)				
Cantril's ladder	0.43	[(0.39)–(0.48)]		
EQ-5D	0.63	[(0.58)–(0.67)]	0.34	[(0.28)–(0.40)]
**Subgroups by study setting**				
**Primary care** (N = 11,892)				
Cantril's ladder	0.41	[(0.33)–(0.48)]		
EQ-5D	0.60	[(0.52)–(0.67)]	0.31	[(0.21)–(0.41)]
**General population** (N = 2,221)				
Cantril's ladder	0.53	[(0.51)–(0.56)]		
EQ-5D	0.71	[(0.68)–(0.74)]	0.43	[(0.35)–(0.50)
**Hospital** (N = 1,534)				
Cantril's ladder	0.43	[(0.35)–(0.51)]		
EQ-5D	0.57	[(0.51)–(0.62)]	0.29	[(0.25)–(0.34)]

Notes:

TOPICS-CEP is a HR-QOL tool, with a range of 0 to 10.

Cantril’s ladder is a genral QOL tool, with a range of 0 to 10.

EQ-5D is a 5-dimensional HR-QOL tool, range -0.33 to 1.00.

### Known group validity

Table 3 illustrates the association between TOPICS-CEP scores and sample characteristics. In line with our expectations, higher mean TOPICS-CEP scores were found in older adults who were married, lived independently and had a higher education level, respectively. Moreover, the mean TOPICS-CEP scores were higher in the persons without dementia, depression and dizziness with falls, respectively. Furthermore, Table 3 illustrates the relationships between TOPICS-CEP scores and sample characteristics adjusted for gender and age. The parameter estimates of marital status and education level remained significant (P-values < 0.05) after adjustments; however, these exceeded the 15% threshold of change. Thus, for example the average difference between TOPICS-CEP scores of persons who were married or cohabiting versus those who had a deceased partner was still significantly different, however the difference between the scores decreased from 0.37 to 0.08. Furthermore, the parameter estimate of living independently with others was no longer significant after adjustment for gender and age. Without adjustment, the average difference TOPICS-CEP scores of persons living independently alone versus living independently with others were 0.19 points and with the adjustment the difference was 0.01 point. When stratified by subgroup similar results were found (data not shown).

**Table 3:** The associations between TOPICS-CEP scores and sample characteristics for the complete study sample and stratified by subgroup.

	Complete sample				Subgroups by study setting					
			Adjusted for gender and age		Primary care		General population		Hospital	
	Estimates	95% CI	Estimates	95% CI	Estimates	95% CI	Estimates	95% CI	Estimates	95% CI
**Age**										
***78 year old***^1^	7.27	[7.05; 7.49]			7.29	[6.99; 7.57]	7.50	[6.95; 9.04]	7.08	[5.66; 8.5]
Per additional year	0.03	[0.02; 0.04]			0.04	[0.04; 0.04]	0.06	[0.05; 0.07]	0.04	[0.03; 0.05]
**Gender**										
***Male***^1^	7.56	[7.32; 7.8]			7.51	[7.21; 7.82]	7.99	[7.45; 8.53]	7.47	[6.08; 8.86]
Female	-0.39	[-0.43; -0.36]			-0.34	[-0.38; -0.3]	-0.48	[-0.58; -0.38]	-0.55	[-0.67; -0.43]
**Marital status**										
***Married / Cohabiting***^1^	7.50	[7.26; 7.74]	7.19	[6.97; 7.42]	7.45	[7.14; 7.76]	7.96	[7.43; 8.48]	7.42	[6.15; 8.7]
Partner deceased	-0.37	[-0.41; -0.34]	-0.08	[-0.13; -0.04]	-0.29	[-0.33; -0.24]	-0.62	[-0.73; -0.52]	-0.53	[-0.66; -0.4]
Other	-0.22	[-0.28; -0.16]	-0.12	[-0.18; -0.06]	-0.17	[-0.24; -0.1]	-0.34	[-0.51; -0.19]	-0.40	[-0.59; -0.2]
**Living arrangements**										
***Independent alone***^1^	7.37	[7.14; 7.59]	7.27	[7.05; 7.49]	7.32	[7.01; 7.64]	7.75	[7.32; 8.17]	7.01	[5.63; 8.4]
Independent with others	0.19	[0.16; 0.23]	0.01	[-0.03; 0.05]	0.16	[0.12; 0.2]	0.21	[0.1; 0.32]	0.40	[0.28; 0.52]
Dependent	-1.01	[-1.08; -0.95]	-0.86	[-0.94; -0.82]	-0.78	[-0.87; -0.7]	-1.32	[-1.44; -1.19]	-1.03	[-1.29; -0.78]
**Education level**										
***Primary school***^1^	7.13	[6.88; 7.37]	7.02	[6.8; 7.25]	7.15	[6.84; 7.46]	7.32	[6.79; 7.88]	6.94	[5.59; 8.3]
Secondary school	0.27	[0.23; 0.31]	0.17	[0.13; 0.21]	0.23	[0.18; 0.27]	0.45	[0.34; 0.55]	0.29	[0.15; 0.43]
University	0.44	[0.39; 0.5]	0.30	[0.24; 0.35]	0.38	[0.32; 0.45]	0.76	[0.61; 0.92]	0.43	[0.26; 0.61]
**Dementia**										
***No***^1^	7.43	[7.1; 7.77]	7.23	[6.93; 7.54]	7.42	[7; 7.83]	7.88	[7.2; 8.56]	7.18	[5.46; 8.89]
Yes	-1.13	[-1.21; -1.04]	-1.04	[-1.13; -0.95]	-0.86	[-0.96; -0.75]	-1.62	[-1.8; -1.44]	-1.75	[-2.41; -1.08]
**Depression**										
***No***^1^	7.42	[7.15; 7.71]	7.25	[6.99; 7.51]	7.44	[7.09; 7.78]	7.78	[7.11; 8.47]	7.25	[5.8; 8.69]
Yes	-1.16	[-1.21; -1.1]	-1.16	[-1.22; -1.11]	-1.11	[-1.18; -1.05]	-1.44	[-1.61; -1.27]	-1.28	[-1.51; -1.06]
**Dizziness with Falls**										
***No***^1^	7.49	[7.2; 7.75]	7.29	[7.04; 7.54]	7.47	[7.13; 7.81]	7.85	[7.22; 8.49]	7.33	[5.84; 8.82]
Yes	-1.07	[-1.11; -1.01]	-1.01	[-1.05; -0.96]	-1.05	[-1.1; -0.99]	-1.18	[-1.32; -1.05]	-1.15	[-1.31; -0.99]

^1^References are in italic bold, for example: Mean TOPICS-CEP Male = 7.56, Female = (7.56–0.39) = 7.17

## Discussion

The purpose of this study was to determine convergent and known group validity of TOPICS-CEP in a large and heterogeneous sample of persons aged 65 years and older. Preference-weighted composite endpoints such as TOPICS-CEP have the potential to facilitate comparative effectiveness research, thus it is important to establish the validity of these kinds of endpoints prior to their use in the population of interest.

In this current study, TOPICS-CEP was able to accurately represent the heterogeneous composition of the overall study population. TOPICS-CEP scores obtained covered most of the entire current score range of the index and there were no floor or ceiling effects found in the total sample nor in the subsample taken from general population, primary care or hospital settings. This is important for its performance as an outcome measure. At the same time, the EQ-5D utility scores showed considerably larger ceiling effects in the general population sample. The most plausible reason why this specific subgroup exhibited this effect would be that the persons from the general population sample were less frail compared to those from the primary care and hospital sample.

Our correlation analyses revealed significant associations between TOPICS-CEP score versus Cantril’s ladder and EQ-5D utility score. The stronger correlation between TOPICS-CEP and EQ-5D indicates that the TOPICS-CEP measures important aspects of health. As expected, the correlation between TOPICS-CEP and Cantril’s ladder was moderate because the two outcomes measure different concepts. Moreover, our findings supported our hypothesis that there would be a strong correlation between TOPICS-CEP components and the EQ-5D dimensions.

TOPICS-CEP scores adhered expected patterns across marital status, living arrangements, and education level. Additionally, TOPICS-CEP was able to distinguish subjects who had dementia, depression, and dizziness with falls even when adjusted for age and gender. These findings further support the overall validity of the tool.

Our results indicate that there were no floor or ceiling effects for TOPICS-CEP in the different settings. However, similar to other studies we found a ceiling effect for the EQ-5D utility score as the percentage of persons with the highest possible EQ-5D utility score of 1.00 exceeded the 15% threshold. These ceiling effects may be due to a small range of responses (3 levels per item).[26, 27]

A major strength of the study is that it highlights that TOPICS-CEP is less prone to floor and ceiling effects. This is critical since this is an issue for other measures such as the EQ-5D. This may be in part because TOPICS-CEP covers a wider range of domains.

The advantage of TOPICS-CEP is that it is the first preference-weighted quality indicator available specifically designed to assess and compare the outcomes of interventions in elderly care. Previous studies have used generic instruments such as the EQ-5D to assess the effect of interventions on health outcomes. However, these are generic health status measurement scales, which all use a number of items that are not appropriate for older subjects, while specific elements most relevant for older persons are not addressed at all. TOPICS-CEP may be of great value for quality improvement in the elderly care. By using preference-weighted outcome measures the desirability of health outcomes are considered. These kind of measures are distinct from health status instruments, because they characterize how health outcomes are valued as a whole based on the values of relevant respondents. TOPICS-CEP reflects on average the value of interventions according to the Dutch population aged 65 years and older. By reporting these values, quality may be well monitored and quality improvement driven.

There are several limitations to consider. Even though a large heterogeneous sample was used to validate TOPICS-CEP, the pooled dataset is not representative of the general population aged 65 and older in the Netherlands. Sampling frameworks varied across individual studies included in TOPICS-MDS some were based on a random sample, though many targeted vulnerable or disease-specific subpopulations. Although distributions of gender, marital status and institutionalization (observed in TOPICS-MDS) broadly reflect the Dutch general population aged 65 years and older, this does not imply that the data set is nationally representative. However, whereas representativeness is crucial for descriptive studies, this is not necessarily true when examining causal mechanisms. Greater emphasis should be placed on identifying and controlling for confounding variables. Thus, despite the over-representation of certain subpopulations, TOPICS-MDS still serves as a rich resource for the validation of TOPICS-CEP. Secondly, TOPICS-CEP has been validated in a sample of the Dutch population but it has not been reevaluated in other (diverse) study populations. Thirdly, additional research is required to examine other important properties of TOPICS-CEP, such as minimal clinically important difference and the sensitivity to detect change. For these reasons, longitudinal validation would be beneficial and are currently under investigation.

In conclusion, preference-weighted multi-faceted endpoints have the potential to facilitate comparative effectiveness research that incorporates patient preferences. This study supports that TOPICS-CEP is a good option for researchers who need an outcome measure to assess important outcomes for older persons even when it is across a range of differently functioning subpopulations. TOPICS-CEP is a robust measure which can potentially be used in broad settings to identify the effect of intervention or of prevention in elderly care. It deserves further spread as the various outcome domains included in the measure are of great importance to the older population. It is important to note that although caregivers’ preferences were included in the development of TOPICS-CEP, this tool was designed to measure HR-QOL in older persons. However, TOPICS-MDS includes a separate care-related QOL instrument for caregivers, the Carer-QOL. Validation work for this instrument has already been performed.[28]
