# Correction: Examining the construct and known-group validity of a composite endpoint for The Older Persons and Informal Caregivers Survey Minimum Data Set (TOPICS-MDS); A large-scale data sharing initiative

**DOI:** 10.1371/journal.pone.0196316

**Published:** 2018-04-23

**Authors:** Cynthia S. Hofman, Jennifer E. Lutomski, Han Boter, Bianca M. Buurman, Anton J. M. de Craen, Rogier Donders, Marcel G. M. Olde Rikkert, Peter Makai, René J. F. Melis, TOPICS-MDS research consortium

The Data Availability statement for this paper is incorrect. The correct statement is: The data sets generated and/or analyzed during the current study were derived from the The Older Persons and Informal Caregivers Survey Minimum DataSet (TOPICS-MDS) repository. Public sharing of data is restricted per the Data Use Agreement. Data are available by applying to the TOPICS Project Group, who may be contacted at topics-mds@radboudumc.nl and after signing a Data Usage Agreement form, available at http://topics-mds.eu. The authors confirm that they did not have any special access privileges that others would not have.

## References

[1] Hofman CS, Lutomski JE, Boter H, Buurman BM, de Craen AJM, Donders R, et al (2017) Examining the construct and known-group validity of a composite endpoint for The Older Persons and Informal Caregivers Survey Minimum Data Set (TOPICS-MDS); A large-scale data sharing initiative. PLoS ONE 12(3): e0173081 https://doi.org/10.1371/journal.pone.0173081 28296910 10.1371/journal.pone.0173081 PMC5351849

